# Perspectives for integration into the local health system of community-based management of acute malnutrition in children under 5 years: a qualitative study in Bangladesh

**DOI:** 10.1186/1475-2891-13-22

**Published:** 2014-03-20

**Authors:** Camille Eric Kouam, Hélène Delisle, Hans J Ebbing, Anne Dominique Israël, Cécile Salpéteur, Myriam Aït Aïssa, Valery Ridde

**Affiliations:** 1 TRANSNUT - (WHO Collaborating Centre on Nutrition Changes and Development), Department of Nutrition, Faculty of Medicine, University of Montreal, 2405 Chemin de la Côte Sainte-Catherine, Montreal, Quebec H3T 1A8, Canada; 2 Nutrition & Health Service, Action Against Hunger France (ACF-France), House - 20, Rd- 117, Gulshan 2, Dhaka 1212, Bangladesh; 3 Nutrition & Health Service, Action Against Hunger France (ACF-France), 4 rue Niepce - 75662 PARIS CEDEX 14, Paris, France; 4 School of Public Health, CRCHUM, Faculty of Medicine, University of Montreal, Saint-Antoine Tower, 850 Saint-Denis, 3rd Floor, Room S03-462, Montreal, Quebec H2X 0A9, Canada

**Keywords:** Acute malnutrition, Under-5 children, Community-based management of acute malnutrition, Preparedness, Health system, Bangladesh

## Abstract

**Background:**

Acute malnutrition is a major cause of death among under-five children in low- and middle*-*income countries. United Nations agencies recommend the integration of community-based management of acute malnutrition (CMAM) into the local health systems for sustainability. The objective of the study was to assess the preparedness of the health system to implement CMAM targeting children under-five years in two sub-districts of Bangladesh.

**Methods:**

The assessment was performed through direct observation of 44 health centres, individual interviews of seven policy makers, three donors, four health and nutrition implementing partners, 29 health workers, and review of secondary data. Assessment themes, derived from the WHO six Building Blocks, were nutrition governance, nutrition financing, health service delivery, human resources, equipment and supply, referral, monitoring and supervision mechanism. They were subdivided into 16 criteria. Findings were compared with CMAM operational recommendations according to WHO, Valid International and Food and Nutrition Technical Assistance guidelines.

**Results:**

The government of Bangladesh has developed inpatient and outpatient CMAM guidelines, and a policy offering free-of-charge health care for under-five children. Nutrition coordination was not under full government leadership. Most of funds (74%) dedicated to CMAM were provided by donors, for short-term interventions. Of the total 44 health centres assessed, 39 (88.6%) were active, among which 4 (10.2%) delivered inpatient services, 35 (89.8%) outpatient services, and 24 (61.5%) outreach services. These were regarded as opportunities to include CMAM activities. There were 48.9% vacant positions and the health workers were not trained for management of acute malnutrition. Equipment and supplies did not meet the operational recommendations for management of acute malnutrition.

**Conclusion:**

Implementing CMAM through the health centres of both sub-districts would warrant progressive strengthening of the overall health system in the light of identified barriers. A short term strategy would consist of strengthening government coordination of nutrition interventions, exploring additional funding sources, equipping and supplying functional health centres, training health workers and actively involving community health workers to cope with health facility staff shortage. A mid-term strategy would consist of securing permanent funding for CMAM, rehabilitating non-functional health centres, attracting and retaining health workers in rural areas.

## Background

Globally, undernutrition, which refers to both protein-energy malnutrition and micronutrient deficiency, is the cause of around 3.1 million child deaths annually in low- and middle*-*income countries [1]. Protein-energy malnutrition in children is clinically classified as marasmus (severe thinness), kwashiorkor (bilateral pitting oedema), and marasmic kwashiorkor (mixed condition)^a^. Three anthropometric indices are used to define child nutritional status: weight-for-height, height-for-age, and weight-for-age. For any one of these indices, malnutrition is defined as a z-score below -2.0 [2]. There are two forms of acute malnutrition/wasting:

(1) Severe acute malnutrition (SAM), defined as weight-for-height below -3.0 z scores of the median World Health Organization (WHO) standards in children 6–59 months of age, and/or mid-upper arm circumference (MUAC) less than 11.5 cm**,** and**/**or the presence of bilateral pitting oedema;

(2) Moderate acute malnutrition (MAM), defined as weight-for-height ≥ -3.0 z and < -2.0 z scores, or MUAC ≥ 11.5 cm and < 12.5 cm and no oedema.

Approximately 19 million children under 5 years are affected by SAM and 33 million by MAM [3,4]. An estimated 2 to 2.5 million of them die annually, of which 0.5 to one million deaths are attributed to SAM [5-7]. Acute malnutrition is the result of an acute decrease in food intake often combined with illness, anorexia, poor appetite, and sometimes medical complications, leading to rapid weight loss or failure to gain weight. Children suffering from this condition have a high mortality risk; however, the situation is reversible with treatment of medical complications and refeeding in a short period of time. Stunting or chronic malnutrition results from inadequate nutrition, care and health, over long period of time, leading to failure of linear growth and poor development. It does not usually pose an immediate threat to life but it is associated with chronic disease risk in the long-term [8-11].

Important achievements have been observed in the management of acute malnutrition over the last decade, particularly with the development of ready-to-use therapeutic foods (RUTF). The use of RUTF has facilitated decentralized ambulatory management of acute malnutrition and has promoted a community approach called community-based management of acute malnutrition (CMAM). CMAM includes four components: (1) *Community outreach* for early detection and referral of malnourished children, follow up through home visits, sensitisation and mobilisation of the community, (2) *Inpatient treatment services* for inpatient care of SAM children with medical complications, (3) *Outpatient treatment services* for ambulatory care of SAM children without complications, and (4) *Services for management of* MAM children [12,13]. CMAM has gained widespread acceptance and is now the preferred model for selective refeeding in different contexts [14-16]. United Nations agencies recommend its integration into local health systems for sustainability, that is taking into account all elements which can influence the implementation of an intervention through the regular health system, while planning CMAM [7,17,18].

In Bangladesh, 34.7% of under-five children are underweight and 48.6% are stunted. The prevalence of global acute malnutrition (GAM) is 13.5%, representing about 2.2 million children, of which 10.1% suffer from MAM and 3.4% (about 500,000 children) suffer from SAM at any one time [19,20]. Malnutrition is involved in half of deaths of under-five children as in many developing countries [1,21-24]. Despite the considerable number of nutrition interventions carried out by non-government organisations (NGOs) in recent years to address malnutrition in the country, acute malnutrition remains an important issue because many projects collapse as soon as NGOs depart from the intervention area or when funding ends [25-27]. The International NGO *Action Against Hunger France* (ACF-France) whose mandate is to address undernutrition and all its direct and underlying causes, arrived in Bangladesh in September 2007, in the context of flood emergency response. The NGO implemented CMAM in the refugee camps of the two sub-districts of Ukhiya and Teknaf (located in the rural area of south-east Bangladesh), and advocated for the implementation of the approach to the government and other partners in the country. The other partner NGO in the two sub-districts was MSF-Holland, implementing health interventions in the camps. In 2009, ACF-France conducted a nutritional anthropometric survey among under-5 children living in the host communities of Ukhiya and Teknaf. The prevalence of acute malnutrition was very similar to national rate, with a GAM prevalence of 11.9% and 14.3% respectively, and a SAM rate of 2% in both sub-districts [unpublished observations]. It was felt relevant to implement CMAM in these sub-districts.

**Figure 1:**
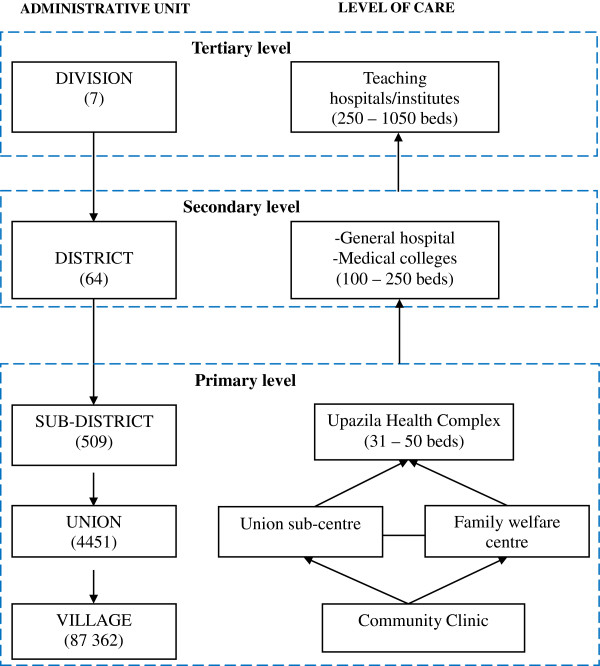
Link between the administrative structure and the health system in Bangladesh.

Bangladesh is divided into seven administrative regions called divisions, each of which is made of 64 districts, subdivided into 509 administrative sub-districts or Upazilas. The Upazilas in turn are subdivided into 4451 Unions, under which there are around 87362 villages. The public health system pyramid follows the country’s administrative structure. The primary level of the health system is located at sub-district, the secondary level at district, and the tertiary level at division and national capital (Figure 1). *The primary level* is organised around one Upazila health complex (UHC) which is the first level referral hospital for the rural population. The hospital has 31 to 50 beds. It is completed at Union level by the Union sub-centres, the family welfare centres and the community clinics. *The secondary level* of the health system is represented by the general hospital with 100 to 250 beds and the medical colleges serving a group of districts (semi-urban areas) and providing inpatient, outpatient, laboratory and imaging services. *The tertiary level* is represented by teaching hospitals/institutes with 250 to 1050 beds, located at division and national levels (urban areas). They mainly offer specialised health care services.

The Ministry of Health and Family Welfare (MOHFW) is responsible at the national level for policy formulation, planning and decision making. Its policies are implemented through four executive authorities, namely: the Directorate General of Health Services, the Directorate General of Family Planning, the Directorate of Nursing Services and the Directorate of Drug Administration. Next to the public sector, there are private hospitals and health centres in rural and urban areas at each level of the health system, mainly managed by NGOs.

In line with the international recommendations to integrate CMAM into the local health system, this study was conducted with the aim to assess the preparedness of the health system and the community to take on CMAM targeting under-5 children in the two sub-districts of Ukhiya and Teknaf in Bangladesh. This article addresses only the health system appraisal. The community assessment will be the topic of another article.

## Methods

### Study settings and design

The qualitative exploratory case study took place from December 2009 until April 2010, at the national level and in the two sub-districts of Ukhiya and Teknaf. The WHO six Building Blocks of the health system, namely *leadership and* g*overnance, health financing, health service delivery, human resources, health information systems, and monitoring and supervision *[28], were adapted to public health nutrition to assess the health system preparedness to integrate CMAM. The six adapted themes were subdivided into 16 subthemes or criteria, determined according to minimum requirements for sustainable CMAM implementation in different contexts, as described in different publications and guidelines. These themes and criteria guided data collection (Table 1):

(1) *Nutrition governance:* related to the existence of a national nutrition policy, nutrition guidelines, and functioning coordination mechanism for nutrition interventions implemented in the country. Nutrition is central for human, social, and economic development, therefore it should be a national priority, which translates into the adoption and promotion of policies, programmes and guidelines [7,16,29].

(2) *Nutrition financing:* existence of a child health and nutrition funding policy, and available funds dedicated to CMAM, coming from national and international sources. Ensuring a free of charge health service for under-5 children, along with permanent funding commitment for CMAM are important elements for sustainability of the intervention [7,16].

(3) *Health and nutrition service delivery:* inpatient, outpatient and outreach activities delivered by the health centres. The objectives of inpatient treatment of malnourished children are to control medical complications and to initiate nutritional rehabilitation on a 24-hour basis. Inpatient management requires dedicated space and beds for malnourished children, a well- equipped kitchen, play area and toys, availability of medicines and therapeutic milks. Dehydration, infections, hypothermia, hypoglycaemia and anaemia are frequent complications that should be controlled in the hospital before transferring the child for outpatient treatment and follow-up [30,31]. SAM children without complications and MAM children should be managed on an ambulatory basis. The family has to collect the RUTF or supplementary foods weekly or biweekly, along with routine medicines [13,30]. Community outreach in CMAM generally consists of mobilisation and sensitisation of community members, active case finding, referral and home visits for follow-up [13,30].

(4) *Human resources:* available staff providing health and nutrition care to malnourished children in the health centres. Their number, motivation, and qualifications for malnutrition management were assessed. According to the Humanitarian Charter and Minimum Standards in Humanitarian Response (SPHERE) [32] and WHO inpatient guidelines [31], one health staff should be in charge of 10 children hospitalised for SAM with complications. For outpatient management of SAM without complications, there should be one health staff in charge of 20 children as per the Valid international Guidelines [13]. In the absence of criteria for determining the required number of staff for MAM management, the same criterion as for outpatient management of SAM children was used in this study, with the assumption that a same number of MAM and SAM children can be managed by one staff. Staff working in the functional health centres should be trained for management of acute malnutrition [7], and they can be motivated through various incentives or good working conditions [30].

(5) *Equipment and supply:* available material in health centres for delivering health and nutrition services to under-5 children. This consists of facilities such as consultation, hospitalisation and storage space, kitchens, toilets, electricity, drinking water source, and play areas. These physical facilities should be equipped with medical and anthropometric materials, and supplied with essential medicines, RUTF and supplementary foods [13,30-32].

(6) *Referral, monitoring and supervision mechanism:* flow of malnourished children between health centres, information flow from the bottom to the top of the health system pyramid, and vice versa, as well as regular field monitoring of activities and supervision of health staff [16,30].

**Table 1:** Criteria/minimum requirements determined for assessing preparedness to integrate CMAM into the health system

**Themes**	**Criteria**
**Nutrition governance**	1. Existence of a government child health/nutrition policy
	2. Inpatient and outpatient CMAM guidelines developed and disseminated
	3. Nutrition coordination mechanism chaired by the Ministry of health from national to district levels
**Nutrition financing**	4. Existence of a funding mechanism/policy for child health/nutrition
	5. Existence of funds allocated to nutrition and CMAM
**Health and nutrition service delivery**	6. Existence of functional health centres
	7. Provision of inpatient health and nutrition services
	8. Provision of outpatient health and nutrition services
	9. Provision of outreach health and nutrition services
**Human resources**	10. Health staff trained on CMAM and sufficient number available for child health and nutrition service delivery
	11. Motivating working conditions for health workers
**Equipment and supply**	12. Inpatient health centres have a paediatric ward, sufficient equipment, storage facilities, essential medicines and therapeutic foods
	13. Outpatient health centres have sufficient equipment, storage facilities, essential medicines and therapeutic foods
**Referral, Monitoring and supervision mechanism**	14. Functional referral mechanism between health centres
	15. Existence of a health/nutrition reporting mechanism
	16. Regular field monitoring and supervision

### Data collection techniques and tools

Three data collection techniques were employed, namely: individual interviews, direct observations, and secondary data review. Secondary data review helped to gather available information on the assessment themes, while direct observation at health centre level provided complementary information, further strengthened by data collected through individual interviews for triangulation. These techniques were applied by the main study investigator, who was assisted by a translator and three transcribers trained on study objectives and methodology.

### Individual interviews

Individuals involved in child health and nutrition programme design and implementation in the country were the main people interviewed, because they were the most able to provide useful information on the study themes. The selection procedure was purposive, using a snowball sampling approach, which is a non-probability technique where existing study subjects recruit future subjects from among their acquaintances [33,34]. Respondents included policy makers, donors (Development Partners, including bilateral and multilateral agencies, and development banks), health and nutrition implementing partners and health and nutrition workers. In the two sub-districts, available health workers who agreed to participate were interviewed. Topics covered during the interviews were derived from the study themes. Questions pertaining to *nutrition governance, nutrition financing, and health and nutrition service delivery* targeted policy makers, donors and implementing partners, while those on *human resources, equipment and supply, referral, monitoring and supervision mechanism* were directed toward health and nutrition workers. Interview guides were developed for the purpose, and initially translated from English to Bangla, then from Bangla to English by two independent translators, in order to ensure accuracy and reliability of the translation. Each interview guide was further pre-tested with three subjects and refined accordingly. The interview sessions, conducted by the main study investigator, were held in English and in Bangla when needed with the assistance of the translator. Interviews were carried out until saturation of content, which occurs when further interviews do not add new information to the findings and become merely redundant [35]. Interviews were audiotaped.

### Direct observation

The 44 existing primary health centres in the two sub-districts were observed to collect information on *health and nutrition service delivery, equipment and supply.* For *health and nutrition service delivery*, the observations focused on infrastructure, facilities, and the performed activities. Regarding *equipment and supply*, the observations focused on materials for medical examination, for anthropometric measurements, for laboratory tests, and on medicines and therapeutic foods. A check-list was developed for this purpose. It was pre-tested in the nutrition service of one of the hospitals and was refined accordingly. The observations were performed by the main study investigator. The health staff was not informed beforehand of the observation, and they were not aware of being observed in their activities.

### Secondary data review

Available official documents pertaining to the national health system and to child health and nutrition were collected at national, district and sub-district levels. They were collected from official sources including the Ministry of Health and Family Welfare, the Institute of Public Health Nutrition, United Nations Children’s Fund (UNICEF), the World Food Programme (WFP), the WHO, NGOs, health inspectors, health workers and local health administrative officers. The reviewed documents included the health system profile, the administrative structure and its functioning, the demographic and health surveys, the government child health policy and strategy, the nutrition guidelines, the annual health reports, and published articles and documents on the nutritional situation of the country. The review of a total of 30 documents helped to get an overview of the national nutrition policies and programmes, as well as insights on the administrative structure and the health system pyramid.

### Data management and analysis

The recorded interviews were transcribed verbatim into English and transferred along with the documents reviewed in the Qualitative Data Analysis software (QDA-Miner 4.0; developed by Provalis Research in 2011, Montreal, Canada) [36] for coding. Data collected through the observation check-lists were entered on Excel sheets in order to compute frequencies. The analysis was thematic, that is, data collected through various techniques were grouped and summarized under each study theme [37]. In order to determine the required staff to operate CMAM in the government health facilities, health workers considered in the analysis were those who had an appropriate training in health and/or nutrition. These included medical officers, medical assistants, senior staff nurses, assistant nurses, pharmacists, sub-assistant community medical officers, health assistants, family welfare visitors and family welfare assistants.

A *normative approach* was employed by two analysts (the main study investigator and another co-author) to interpret the findings. This consisted of first summarizing the findings under each theme, then assessing them with the identified operational norms (when existing) or recommendations for CMAM implementation according to the Valid International [13], FANTA [30], WHO [31] and the SPHERE [32] guidelines.

### Ethical considerations

The study was approved by the Bangladesh Ministry of Health and Family Welfare through the Institute of Public Health Nutrition, and by the Ethical Committee of the Faculty of Medicine, University of Montreal, Canada. Informed consent was obtained from all participants. Their names were removed during analysis to ensure confidentiality.

## Results

A total of 43 respondents were interviewed. They included seven policy makers, three donors, four health and nutrition implementing partners and 29 health and nutrition workers. Data collected through individual interviews, along with those obtained from direct observations and review of documents are presented in this section according to the predetermined themes and criteria.

### Nutrition governance

#### *Government child health/nutrition policy*

Improvement of nutritional status of children has been a priority of the government of Bangladesh for many years, and the MOHFW has developed successive policies in this regard. The Bangladesh integrated nutrition programme was the first comprehensive nutrition programme implemented in the country from 1996 to 2002, aimed at reducing the incidence of low birth weight and malnutrition in children. From 2002 onwards, the intervention was continued as a national nutrition project (NNP) funded by the World Bank and operated by local NGOs. One of the six objectives of the NNP was to reduce the prevalence of severe and moderate malnutrition in young children. In 2006, the NNP was integrated into the health, nutrition and population sector programme (HNPSP), a policy instrument for improving the nutritional status of children. The programme was implemented through several contracted NGOs. It faced coordination, monitoring and evaluation challenges, and it did not fulfil the needs of SAM children. In light of this experience, the MOHFW revised the policy so that nutrition interventions would be implemented through the national health system. This policy change was reflected in the most recent health, population and nutrition sector development programme (HPNSDP 2011–2016). The NNP activities are to be operationalised as national nutrition services (NNS) delivered through the national health centres, and CMAM is to be one of the NNS interventions [25].

#### *Inpatient and outpatient CMAM guidelines development and dissemination*

The guidelines for inpatient management of SAM were developed in 2008, but they were not yet disseminated at the time of the study. These guidelines recommend facility-based treatment of SAM children with therapeutic milks F-75 and F-100, and with local equivalents called *‘Khichuri, milk suji, and halwa’*, prepared from local ingredients^b^. Training modules were under development at the time of the study and were designed for senior nurses and doctors in hospitals. As CMAM training was not included in the curriculum of medical schools, the MOHFW in collaboration with implementing partners planned to organize the training of authorized health professionals as ‘Master trainers’ for other health staff in the various health centres. Inpatient guidelines were finalised in March 2012 and disseminated with associated job aids and tools in the health centres. The MOHFW has organised the training of Master Trainers since then, and by the last quarter of 2013, the treatment of SAM children was implemented in 39 inpatient facilities across the country (including the two sub-districts assessed).

Outpatient guidelines were not yet developed at the time of the study. The MOHFW was planning to adapt the global CMAM approach to the local context, recommending the use of ready-to-used foods made from locally-available ingredients, for outpatient management of SAM children without complications and MAM management. These recommendations were based on scientific evidence showing the acceptability and effectiveness of locally prepared foods in facility and community-based treatment and prevention of severely and moderately malnourished children in Bangladesh [38-45]. Outpatient treatment guidelines were issued in September 2011, and disseminated. Nutritional Treatment (NT), a locally prepared and pre-packaged oil-based, energy-dense and mineral/vitamin fortified nutritious food, is since recommended for outpatient treatment of SAM children without complications^c^.

Moreover, the MOHFW, through the International Centre for Diarrheal Disease Research, Bangladesh, planned to conduct from 2012 on, operational research comparing the acceptability, effectiveness and cost-effectiveness of peanut-based RUTF with ready-to-use foods prepared from local ingredients, for outpatient treatment of SAM children. These studies are still on-going as of 2014, and the conclusions are awaited.

#### *Nutrition coordination mechanism*

A national nutrition working group, in operation since December 2004, coordinates all stakeholders (donors and NGOs) implementing nutrition activities in the country. This working group, operating from national to district levels and chaired by UNICEF, holds monthly meetings and disseminates information among its members. Although UNICEF and the other participating members would have appreciated more government involvement, the MOHFW has not been fully part of the nutrition working group, which operated in parallel fashion with the government national nutrition task group operating within the health, nutrition and population sector programme. Thus, strengthening collaboration among the government and partners from national to district levels is an issue that was raised by interviewees.

*“It is appreciated that NGO activities are implemented in different areas of Bangladesh. But I want to know if the Bangladesh government estimates that the NGOs are running their programs with string line of government policy…All the NGOs and government should sit together and find a good and proper co-operative way to work for health and disaster situation management that will be more effective and sustainable for people…”* [Decision-maker, MOHFW, Dhaka].

### Nutrition financing

#### *Child health/nutrition funding mechanism and policy*

There was a sector-wide approach to nutrition financing, bringing together the government, donors, civil society and other stakeholders. The MOHFW was supported by donors, who accounted for 30% to 40% of total health, nutrition and population sector expenditures over the last ten years [46]. Major contributors were the World Bank, the United Kingdom Department for International Development (DFID), the United States Agency for International Development (USAID) and UNICEF, in addition to 14 other donors. Seven per cent of the governmental health budget was reportedly dedicated to nutrition. Nutrition funds from external sources were available and planned for the coming years. Bangladesh health policy provided a free of charge primary health care for under-5 children, annually funded by the MOHFW.

#### *Funds allocated to nutrition and CMAM*

During the assessment, United Nation agencies including UNICEF, WFP and Food and Agriculture Organisation (FAO) expected to receive USD $8 million from the Millennium Development Fund, while UNICEF expected USD $7 million from the European Union in 2010. These funds aimed at covering activities related to food security and to piloting CMAM for three years. In order to enhance the impact of the CMAM projects, UNICEF also planned to run micronutrient supplementation programmes as a way of combating the high prevalence of anaemia, as well as promoting Infant and Young Child Feeding (IYCF). The expected funds for CMAM were mainly provided by donors, and they were to be shared among different implementing partners for piloting the intervention in priority districts.

The expected funds for 2010 were received and allocated to dedicated nutrition activities and CMAM pilot in the country, including the two sub-districts assessed. The total budget of the current health, nutrition and population sector programme (HNPSP July 2011 – June 2016) amounts to USD $7.7 billion, funded by both the MOHFW (74%) and donors (26%). The National Nutrition Services budget is around USD $192,892,401 (2.62% of the total HNPSP budget), of which around USD $7.7 million (3.89%) is allocated to CMAM (the MOHFW providing 26% and donors 74% of the total amount) [26].

### Health and nutrition service delivery

#### *Functional health centres*

Of the 44 health centres surveyed in the two sub-districts, 39 (88.6%) were active during the assessment period (Table 2). Five (11.4%) community clinics were not functional because of lack of staff. The health centres offered inpatient, outpatient and outreach services.

**Table 2:** Health facilities in the two sub-districts of Ukhiya and Teknaf, Bangladesh, 2010

		**Health facilities**	
**Level of care**	Active*	Not active*	**Total**
Upazila Health Complex (UHC)	2	0	2
Union sub-centre (SC)	7	0	7
Family Welfare Centre (FWC)	6	0	6
Community Clinic (CC)	22	5	27
NGO Health Centres	2	0	2
Total	39 (88.6%)	5 (11.4%)	44 (100%)

*Active: services were delivered during the assessment period; Not active: no services were delivered during the assessment period.

#### *Inpatient health and nutrition services*

Out of the 39 active health centres, four (10.2%) delivered inpatient and outpatient health activities. These were the two Upazila health complexes and two NGO health centres (Table 2). Patients could be hospitalised and monitored on a 24-hour basis.

Services provided in the Upazila health complexes were emergency care, delivery care, contraceptive surgery, limited imaging services and basic laboratory testing. They also offered outpatient services such as antenatal and postnatal care, dental care and expanded programme of immunisation. The imagery service was not provided in one hospital because of lack of a technician to operate the x-ray machine. Performed activities linked to nutrition were essentially the growth monitoring and promotion programme. Nutritional status of children was assessed monthly, using weight-for-age to detect underweight cases. Mothers/caretakers were advised on adequate infant and child feeding and hygiene practices. There was no screening or treatment for acute malnutrition in the two Upazila health complexes.

#### *Outpatient health and nutrition services*

A total of 35 (89.8%) health centres assessed delivered only outpatient services in the two sub-districts (Table 2). These health centres included Union sub-centres, family welfare centres and community clinics. Patients were managed on an ambulatory basis. The Union sub-centres treated common diseases, while the family welfare centres provided antenatal and postnatal care, normal delivery, health education and family planning. Postnatal care included growth monitoring and promotion. The community clinics also conducted immunization and treatment of common diseases, health workers alternatively providing services three days in the clinics and three days in home visits. The main complaint raised by sub-centres health workers were the high workload during rainy seasons because of the dramatic increase of diarrhoea cases and acute respiratory infections in children during that period of the year.

#### *Outreach health and nutrition services*

Out of the 39 active health centres, 24 (61.5%) performed outreach activities, 22 community clinics and the 2 NGO health centres (Table 2). The community clinic health workers, namely a health assistant and a family welfare assistant, alternatively made home visits three days a week to offer preventive services that include immunisation, education on contraceptive methods and birth control, good hygiene, nutrition and health care practices, and distribution of contraceptive pills and condoms. However, screening and managing acute malnutrition were not part of their community health activities.

*“My responsibility is to cover 2 to 3 wards. The nearest ward has 200 to 250 households. There are around 150 households in the other wards…I have to cover all the wards in a month to achieve my target…”* [Community clinic health worker, Ukhiya].

The main challenges raised by these health workers were the difficult access to hilly areas during rainy seasons and the inability to cover all the expected households every month because of large catchment areas in some villages.

### Human resources

#### *Health staff training on CMAM and number of health workers available*

At facility level, a malnourished child can be identified through anthropometric indices and/or clinical examination. In both districts, health staff in charge of health centres, who are usually skilled health workers, could clinically recognise a malnourished child: “*the baby is thin… the face is fatty and watery… he has lost skin… he has anaemia…”* However, they were neither able to make a proper anthropometric assessment of malnutrition as per the national guidelines, nor able to adequately manage malnutrition cases, because of lack of training.

*“We do clinical observation and we guess if the child is malnourished. We give them some B vitamin and iron tablets, and we advise parents to feed the child with nutritious food like khichuri.”* [Sub-centre health worker, Ukhiya].

NGO health centres did not show any shortage of staff. However, there were nearly half vacant posts in the government health facilities of both sub-districts. Family welfare centres showed higher vacancy rate (70.8%) and 46 posts were vacant for Union sub-centres, family welfare centres and community clinics (Table 3).

**Table 3:** Available staff in the active health facilities, Ukhiya and Teknaf sub-districts, Bangladesh, 2010

	**Staff**			
**Level of care**	**Expected**	**Existing**	**Vacant**	**% vacant**
Upazila Health Complex (UHC)	39	14	25	64.1
Union sub-centre (SC)	28	10	18	64.2
Family Welfare Centre (FWC)	24	7	17	70.8
Community Clinic (CC)	54	43	11	20.3
Total	145	74	71	48.9

*“Manpower is the main problem here. According to the government policy, there should be two health workers in one community clinic to cover 6000 persons; but I am the only health assistant here to cover more than 12000 persons…”* [Community clinic health worker, Ukhiya].

*“The medical assistant that was working with me was transferred to another Upazila…The government appointed a new one but he is not willing to join his position here…”* [Sub-centre health worker, Teknaf].

Based on ACF-France nutritional anthropometric surveys conducted in both sub-districts in 2009 using WHO growth standards of 2006, the estimated yearly caseload was 1860 SAM and 10,525 MAM children. Assuming that 5% of SAM children needed inpatient care and 95% outpatient care (on the basis of admission trends in CMAM implemented previously by ACF-France in the refugee camps), the estimated caseload at any one time is 93 SAM children for inpatient care and 1767 SAM children without complications for outpatient management (Table 4).

**Table 4:** Needed staff for inpatient and outpatient SAM and MAM management, according to existing recommendations

	**SAM inpatient**	**SAM outpatient**	**MAM outpatient**
Total malnourished children at any time	93	1767	10,525
Recommended number of children to be managed by one staff	10	20	No standard (20)
Total staff needed	0	88	526
Existing staff	14	60	60
Gap in staff number	0	28	466

Note:

Existing staff for inpatient management are those of Upazila health complexes.

Existing staff for outpatient care are those of sub-centres, family welfare centres and community clinics.

According to the Humanitarian Charter and Minimum Standards in Humanitarian Response (SPHERE) and WHO recommendations for inpatient management of SAM children with complications, the available staff in the Upazila health complexes was adequate in numbers to cover the caseload of SAM children requiring inpatient management. According to Valid International recommendation for outpatient SAM management, 28 additional staff would be necessary to cover the caseload of SAM children without complications. If the 46 vacant positions of outpatient health centres were filled, the recommendation would have been met. However, 466 health employees would be required to properly handle outpatient MAM caseloads (based on the same staff:patient ratio as for outpatient SAM). This is 16 times higher than the 28 additional employees needed for outpatient SAM management, which would not have been met even with the fulfilment of the 46 vacant positions.

#### *Motivation and working conditions of health workers*

According to interviewees, the high vacancy rate in the two sub-districts is not due so much to the lack of trained staff, but rather to the fact that many appointed health workers were not willing to work in these areas because of their *“remoteness”* and the absence of accommodation which is supposed to be provided by the government. Consequently they prefer to stay in the capital or the major cities, or to work for NGOs which provide them with better salaries and working conditions.

Health workers interviewed in the two sub-districts were in favour of implementing inpatient and outpatient CMAM activities along with their regular health activities, as long as they are trained and as the activities are included in their job description by the Ministry of health. At national level, policy makers and implementing NGOs were well aware of the staff shortage situation in the rural areas of the country, and they were favourable to the involvement of more community health workers and community volunteers to make up for the health facility staff shortage.

### Equipment and supply

#### *Paediatric ward, equipment, storage facilities, essential medicines and therapeutic foods procurement in the inpatient facilities*

The two Upazila health complexes had consultation, hospitalisation and storage rooms. Each hospital had a functional water source, as well as latrines and electricity, and there was available space that could be used as play area for children. In addition, kitchen facilities were available, but they were not well equipped. The hospitals did not have paediatric wards and children were hospitalised in the women wards.

Clinical examination materials such as stethoscopes, thermometers and sphygmomanometers were available and in working condition, as well as microscopes for laboratory testing. However the weighing scales and measuring boards were out of order. There were no toys for child stimulation in the hospitals. According to health workers, the Upazila health complexes were regularly supplied with routine medicines, but there were no therapeutic milk ((F-75/F-100) and RUTF, and no medicine kits (antibiotics, oral rehydration solutions, micronutrient supplements) for inpatient management of SAM. None of the hospitals was in a position to provide *khichuri* and *halwa* prepared from local ingredients as per the national guidelines for inpatient management of SAM.

#### *Equipment, storage facilities, essential medicines and therapeutic foods procurement in the outpatient facilities*

The family welfare centres, Union sub-centres and community clinics had consultation rooms, storage rooms and equipment for clinical examination, but they lacked water sources, electricity, latrines and well-functioning equipment for anthropometric measurements. None of the facilities had MUAC tapes available, although national guidelines recommend MUAC measurement as the admission criterion for acute malnutrition in children.

Community clinics did not have good storage facilities. Medicines were generally stored in wooden cabinets exposed to humidity and invaded at times by rodents. Some health workers took medicines home at the end of each working day and took them back to the clinic the next day in order to cope with such inconvenients.

A total of 86% of outpatient health facilities were supplied with routine medicines, but not on a regular basis, and quantity supplied could not meet the demand, as the government provides a fixed amount of money every year to each health centre for medicine supply, irrespective of the caseload.

*“We face lots of problems because of the very little amount of medicines available as compared to the number of patients… I have to ask patients to buy medicines from outside the health centre, but most of them are not happy because they come here to get medicines for free…”* [Sub-centre health worker, Ukhiya]*.*

The availability of medicines in the health centres had an important influence on the attendance. According to health workers, the attendance drastically decreases with medicine shortages and some community clinics are even closed during periods of shortage.

### Referral, monitoring and supervision mechanism

#### *Referral mechanism between health centres*

There was no formal referral mechanism between the health centres and it is therefore not possible to track patients’ movements between them. It was the responsibility of patients to get to the referred health centre/hospital, when sent by the health professional.

*“When I refer patients to the Upazila health complex I cannot follow them up there. They keep in touch with me when they are discharged…If they do not come to me, I can meet with them when I am visiting households in the villages”.* [Community clinic health worker, Ukhiya].

#### *Health/nutrition reporting mechanism*

Health workers in charge of community clinics, Union sub-centres and family welfare centres submitted their monthly activity reports to their supervisor, the health inspector or the family planning inspector in the Upazila health complex. From the Upazilas the reports were transferred to the district where they were tallied, and submitted to the division, then to the national level. Health workers mentioned that they were sometimes recalled to make adjustments in their reports in case of mistakes, but they did not usually get any feedback from the Upazila and district health offices after submitting their final monthly reports. Nutrition indicators were not included in the health information system, and therefore, it was not possible to determine how many children were screened and managed for malnutrition.

#### *Field monitoring and supervision*

Health workers declared being supervised on a monthly basis by the Upazila health inspector. He visited the health facilities, observed the delivery of services, discussed with the health workers about their daily management difficulties and provided practical advice and instructions.

## Discussion

The objective of the study was to assess the preparedness of the health system to take on CMAM in the two sub-districts of Ukhiya and Teknaf. Table 5 presents a synthesis of key findings obtained through the use of the adapted themes and criteria.

**Table 5:** Synthesis of findings obtained from the use of the adapted WHO six building blocks

**Theme**	**Key findings**	
	**Facilitating factors**	**Challenging factors**
**Nutrition governance**	-Nutrition is a priority for the government	-Nutrition coordination not fully under government leadership
	-Child nutrition policy developed	
	-CMAM is part of the nutrition strategic interventions in the country	-CMAM training not yet included in the curriculum of medical schools
	-Inpatient and outpatient CMAM guidelines developed and disseminated	
**Nutrition Financing**	-Primary health care service free of charge for under- 5 children	-Most of CMAM funds provided by donors, and for short term interventions
	-Funds dedicated to nutrition and CMAM available	
*Health and Nutrition Service Delivery*	-Primary health care activities delivered in the health facilities, including growth monitoring and promotion programme	-Screening and management of acute malnutrition not delivered in the health centres, and not included into outreach health activities
	-Outreach health activities performed by community clinics health workers	
**Human resources**	-Available staff adequate in numbers to cover the caseload of inpatient management of SAM children with complications	-Health workers not trained for adequate identification and management of acute malnutrition
		-Insufficient number of health facility staff to handle outpatient SAM and MAM caseloads
		-Health workers not willing to work in rural areas
**Equipment and supply**	-Presence of consultation rooms in the inpatient and outpatient health centres	-Absence of dedicated spaces for children in the hospitals
	-Presence of medical material in good working condition	-Absence or inadequate latrines and water sources
	-Available kitchen facilities in the inpatient health centres	-Lack of anthropometric materials
		-Absence of play areas and toys for children
		-Kitchen facilities not well equipped
		-Inadequate storage facilities
		-Absence of paediatric wards
		-Insufficient supply of medicines
		-Absence of therapeutic and supplementary foods
**Referral, monitoring and supervision mechanism**	-Existence of a functional reporting mechanism	-Absence of a formal referral mechanism
	-Existence of a regular supervision	-Nutrition indicators not included in the health information system

Improving child nutrition is a government priority in Bangladesh, and the national health and nutrition policy fostered CMAM advocacy and potential integration into the health system [26]. The MOHFW considered CMAM as a relevant intervention and developed adapted inpatient and outpatient guidelines. The UN Joint Statement on CMAM states that the development of national protocols is crucial prior training of health workers on a standardised CMAM approach delivered at all levels of the health system pyramid [7,47]. Bangladesh has made a remarkable progress in this regard, treatment of SAM children being implemented in 39 inpatient facilities across the country. However, while moving forward with the issue of CMAM integration in the country, the MOHFW should lead the nutrition coordination process (including the nutrition working group). This would harmonise and strengthen the identification and allocation of available resources for CMAM to priority districts, for better impact.

For CMAM integration to be sustainable, it is essential to have a commitment for long-term funding [7,16]. Available funds dedicated to CMAM at the time of the study were earmarked for short term interventions (3 years). Successful design and implementation of CMAM pilot projects would serve as advocacy instrument for pledging more funds from both government and partners. The free-of-charge medical care for children represents an opportunity for many malnourished children to access health and nutrition services, as most of them generally come from poor families. However, such policy, although internationally supported by some funding agencies [48] and global health actors [49], might be challenging in the context of short-term funding projects. Bangladesh joined the Scaling-Up Nutrition (SUN) movement as an active participant [50], therefore the government and partners should use this opportunity to secure long term funding for CMAM. The Renewed Efforts Against Child Hunger (REACH) initiative [51], disaster risk reduction as well as HIV and AIDS initiatives should also be scrutinised. In addition, options such as participation of private investors, foundations, donor states, solidarity lottery and small tax levy on financial transactions should be examined in order to widen the pool of available funds for nutrition [52].

Services delivered within the hospitals and the health centres of the two sub-districts offered opportunities to include inpatient, outpatient and outreach CMAM activities. For example, services provided in the Upazilla health complexes demonstrated that health workers would be able to manage complications related to malnutrition if they were trained and if the hospitals were rehabilitated, equipped and supplied.

Outpatient services provided in the Upazilla health complexes, Union sub-centres, family welfare centres, community clinics and NGO health centres offered opportunity to integrate outpatient activities for SAM and MAM management. In order to achieve this objective, these health centres would need to be provided with anthropometric and medical materials, storage facilities would have to be improved, a permanent supply of medicines and therapeutic foods would need to be secured, and non-functional health centres would require rehabilitation.

Health assistants and family welfare assistants alternatively visit the households, which is an opportunity to include CMAM outreach in their activities. In this regard, the assistants would have to be trained and provided with mid-upper arm circumference tapes, referral slips and sensitisation materials such as flyers or pamphlets containing Information, Education and Communication (IEC) messages on prevention of acute malnutrition.

In countries like Ethiopia, Sierra Leone, Malawi and Ghana, CMAM activities have been closely linked to antenatal care, IYCF, immunisation, Integrated Management of Childhood Illness (IMCI) and growth monitoring interventions [53-56]. In Mozambique, CMAM is integrated into the reproductive and maternal child health, HIV and AIDS and tuberculosis services, health promotion, and community involvement. In Kenya, most outpatient treatment services for SAM management are located in the Maternal and Child Health clinics [57,58]. The two main facilitating factors in these countries are (1) the leadership of the government for the implementation of primary health care package, in which they have included CMAM activities, and (2) good coordination between the government, NGOs and donors for strategizing integration within the health system and scaling up CMAM services throughout the country [17,59,60]. Similar approach should be considered in the context of Bangladesh for successful inclusion of CMAM activities into the national primary health care package.

Staff shortage would be an important challenge to overcome when planning and implementing CMAM through the health centres of the two sub-districts of Ukhiya and Teknaf. Shortage of health personnel is a recognised issue globally [61]. All countries in Southeast Asia face problems of uneven distribution of health workers, with rural areas often understaffed [62]. The study findings confirmed that health workers prefer to work in the cities and for NGOs because of better working conditions and salaries. This is in accordance with reports from the Asia-Pacific and Africa regions, which indicate that salaries and benefits, together with working conditions, supervision and management, education and training opportunities are factors affecting health worker motivation and retention [63,64]. Proper distribution of health workers, along with assessing appropriate strategies for attracting and retaining them in the rural area would be critical in the context of both sub-districts, prior and during implementation of CMAM [62-65].

One appropriate strategy to offset health facility staff shortage would be the identification, training and involvement of community health workers and volunteers into outpatient SAM and MAM management, since most of the affected children can be successfully treated in the community [30]. A study carried out in Burkina Faso showed that 77% of children suffering for acute malnutrition were successfully managed in the community [66], while CMAM evaluations conducted in Nepal and Pakistan demonstrated that this was the case for more than 80% of children admitted in the programme [67,68]. Recent studies conducted in southern Bangladesh showed that community workers were able to achieve quality care in managing cases of SAM without complications, provided they received good training and regular supervision [69]. The intervention achieved high coverage, low default, high recovery and low mortality rates, and it was cost-effective [70,71]. The approach was also successful in other settings for early detection of malnutrition cases and considerable reduction of death rate [57,72]. Therefore, involving community workers in CMAM implementation in Bangladesh would be an effective strategy to make up for the health facility staff shortage, and would eventually reduce their workload.

The needed staff for outpatient SAM and MAM management was estimated on the basis of standards developed in the contexts of emergency relief interventions, where most of nutrition activities are implemented and managed by NGOs. These standards might not apply in non-emergency contexts when planning the implementation of CMAM through the regular health facilities. Studies should be conducted on the relevance of these standards in such contexts, for example by assessing health workers’ workload during outpatient SAM and MAM management.

The assessment of only two sub-districts and the fact that respondents were selected purposively are two potential limitations of the study. The findings are therefore not representative of the whole health system in Bangladesh, and this may hamper the external validity of the study. There is also a potential source of social desirability bias because interviewees might have answered to questions in such a way that would encourage the study team, ACF and other implementing partners to initiate CMAM in the area. This bias was anticipated and mitigated by triangulating the data collected through three different techniques.

In the absence of standardised criteria and indicators for CMAM integration, the WHO six building blocks were adapted and subdivided into 16 criteria to assess the health system preparedness for CMAM implementation. A systematic review conducted in 2009 used a similar approach to examine the extent and nature of the integration of targeted health programmes into the health system, and it showed that specific interventions are seldom fully integrated into health system functions [73]. In 2012, the use of the WHO building blocks to assess the effects of user fee exemption policies on health system functions gave an idea of what should be expected if such policies did not implement all the required conditions in terms of preparation, planning and complementary measures [74]. In the present study, each criterion provided useful information which helped to make a diagnosis of the health system preparedness, highlighting areas that needed strengthening prior and during implementation of CMAM. This is one of the contributions of the study. Several developments have occurred in the country since the completion of the study, which confirmed the progressive integration of CMAM into the health system. For example, CMAM has been included in the nutrition policy and service delivery, additional funding has been secured, the guidelines have been developed and the health personnel trained, and activities such as hiring more health workers, improving the national health information system, and building and rehabilitating health centres have been planned [25,27]. Additional research conducted both within and outside the Bangladesh context is necessary to sharpen the criteria used in this study.

## Conclusion

The health system assessment demonstrated that there was an enabling environment for CMAM integration in the two sub-districts of Ukhiya and Teknaf, despite identified shortcomings. A short term strategy would focus on strengthening the leadership of the government for nutrition coordination and implementation, identifying additional sources of CMAM funding, equipping and supplying active health centres, training health workers for management of acute malnutrition and involving community health workers and volunteers into outpatient SAM and MAM management. In the mid-term, rehabilitating and equipping non-functional health centres, attracting and retaining health workers in the rural areas, and ensuring long-term funding commitment would be needed for the effective integration of CMAM in the health system in both sub-districts.

## Endnotes

^a^‘Protein-energy’ malnutrition is now referred to simply as ‘malnutrition’ as it is a multi-nutrient deficiency syndrome.

^b^*Khichuri* is a preparation made from rice and lentil paste mixed with soybean oil and boiled vegetables; 100 g provides 140 kcal and 3 g of protein. *Milk suji* is composed of milk powder, rice powder, sugar, soybean oil, with added magnesium chloride, potassium chloride, and calcium lactate. The energy content is 67 kcal/100 mL and the protein content is 1.4 g/100 mL. *Halwa* is a sweet dish consisting of carrots boiled with milk, almonds, sugar, butter, and cardamom; 100 g provides 240 kcal and 5 g of protein.

^c^NT provides 450-550 kcal/100 g; 45-60% of total energy is from fat and 10-12% from protein. For outpatient management of MAM children, mothers/caretakers are encouraged to increase the intake of home food by adding at least 25 kcal/kg/day over and above the energy requirements of a well-nourished child. MAM children living in extremely food insecure conditions would receive a nutritional supplement providing 700–1000 Kcal/child/day, with 25-30% of energy from fat and 10-12% of energy from protein.

## Abbreviations

ACF-France: Action against hunger France; CMAM: Community-based management of acute malnutrition; F75 and F100: Therapeutic milks; FANTA: Food and nutrition technical assistance; GAM: Global acute malnutrition; IYCF: Infant and Young Child Feeding; MAM: Moderate acute malnutrition; MOHFW: Ministry of Health and Family Welfare; MSF: Medecins Sans Frontieres (Doctors without borders); NGO: Non-Government Organisation; NNP: National Nutrition Programme; RUTF: Ready-to-use therapeutic food; SAM: Severe acute malnutrition; SPHERE: Humanitarian charter and minimum standards in humanitarian response; UHC: Upazila health complex; UNICEF: United Nations Children’s Fund; USD: US dollars; WFP: World Food Programme; WHO: World Health Organisation.

## Competing interests

The authors declare that they have no competing interests.

## Authors’ contributions

CEK conceptualised the study protocol and HD contributed to its improvement. CEK implemented the study in Bangladesh, analysed the findings with HJE. CEK wrote the first draft of the manuscript. HD initiated the review of the draft with VR, and all co-authors critically reviewed it. CEK handled the revisions with the support of HD and VR. VR is a Canadian Institute for Health Research New Investigator. All authors accepted the final version of the manuscript.
